# The Value Question in Metaphysics

**DOI:** 10.1111/j.1933-1592.2012.00589.x

**Published:** 2012-06-01

**Authors:** Guy Kahane

**Affiliations:** University of Oxford

## Abstract

Much seems to be at stake in metaphysical questions about, for example, God, free will or morality. One thing that could be at stake is the value of the universe we inhabit—how good or bad it is. We can think of competing philosophical positions as describing possibilities, ways the world might turn out to be, and to which value can be assigned. When, for example, people hope that God exists, or fear that we do not possess free will, they express attitudes towards these possibilities, attitudes that presuppose answers to questions about their comparative value. My aim in this paper is to distinguish these evaluative questions from related questions with which they can be confused, to identify structural constraints on their proper pursuit, and to address objections to their very coherence. Answers to such evaluative questions offer one measure of the importance of philosophical disputes.

Does God exist? Do we have free will? Are there objective moral facts? These are familiar metaphysical questions. Such questions can seem extremely *important*. This might be because they are questions about the most fundamental aspects of the universe. They are about truths that it is valuable to know. But such metaphysical questions are also important because much is at stake in them.

There can be much at stake in them because the answers have important *practical* implications. If we do not have free will, then perhaps we should not hold each other responsible. If God does not exist, we could hardly have reasons to worship Him.

But truths can matter in a more direct way. It might be *worse* if we do not have free will, or if there are no objective moral requirements. Thus for every metaphysical question, we can ask a further evaluative question. Just as we can ask

(1) Does God exist?

we can ask

(2) If God exists, will that be good, and better?

Although vast effort has been spent trying to answer metaphysical questions such as those about God's existence or free will, and although the practical implications of some of these questions are sometimes considered, these further evaluative questions have been almost entirely neglected.^1^

Answers to traditional metaphysical questions tell us what kind of world we inhabit. Answers to these further evaluative questions will tell us how good the world is, and whether it is better or worse than the alternatives.

We can ask these evaluative questions even if we do not have answers to the metaphysical questions that inspire them. If we do not yet know what the world is like, then answering such evaluative questions will tell us how we ought to hope things turn out. And our answers to these evaluative questions will also help us determine the importance of the parallel metaphysical ones. If much is at stake in the question of God's existence—if different answers mean that things are far better or worse—but little is at stake in this way in the question whether numbers exist, then disputes about the latter are, in one obvious sense, less important.^2^

These evaluative questions are seldom raised explicitly. But answers to them are presupposed by common ways of talking and feeling. Theists often describe a Godless universe in bleak terms. Some atheists concur, and lament God's absence. Other atheists spend much effort arguing that naturalism doesn't have such unappealing implications. Similar anxiety is expressed about the prospect that morality is merely a myth. I once heard a philosopher describe compatibilism as ‘second best’, something we learn to accept once we realize that we cannot have libertarian freedom; a libertarian passionately replied that this fails to see just how *awful* it would be if we only had compatibilist freedom.

When people express such attitudes, this typically occurs when they are discussing metaphysical questions, and these metaphysical questions are not clearly distinguished from the further evaluative ones. It is common for thinkers to portray the metaphysical view they hold true to also have superior or at least favourable enough consequences. But even if atheism has bleak implications, this hardly shows that it is not true. Things might not be as good as they might have been. The universe we inhabit might indeed be second best—or worse. What is true is one thing, what is good or better often another.

I will not be offering answers to these evaluative questions about God or free will. My aim here is not to answer such questions, but to distinguish them from other questions with which they are often conflated, to identify structural constraints on their proper pursuit, and to answer objections to their very coherence.

## I. Clarifying the Question

### Questions about Practical Implications

Substantive evaluative questions about the possible outcomes of disputes in metaphysics need to be distinguished, I said, from the metaphysical disputes that underlie them—from questions about metaphysical truth, such as

(1) Does God exist?

They also need to be distinguished from questions about the practical implications of these possible outcomes, such as

(3) If God exists, what difference will that make to our reasons for action?

To the extent that the answer to some metaphysical question would make a genuine practical difference—would make a difference to what we ought to do—then it becomes important to answer it, and to answer it correctly. If we get the metaphysics wrong, we are likely to fail to do what we ought to. If we reject morality by mistake, we might fail to meet our obligations.

But this is not the only way in which answers to metaphysical questions might matter, and not the only thing that could be at stake in them. If morality is an illusion, there are a lot less oughts than we thought. That's a dramatic normative implication. But it's an entirely separate thing to ask whether this would be *better* or *worse*. The noncomparative evaluative question

(2NC) If God exists, will that be a good thing?

and the comparative question,

(2C) Will it be better if God exists, than if He does not?

are very different questions from (3). A world in which our reasons for action are dramatically different might also be a world that is neither better nor worse than the alternative. Practical implications can be driven by differences in value, and they can lead to differences in value, but they need not, and they do not on their own, decide the value of a possible world. Indeed, worlds can greatly differ in value without this making any significant practical difference. And worlds can differ dramatically in what we have reason to do without differing in value in any tangible respect. Questions about value, and about what we ought to do, are different kinds of questions.^3^

### Questions about the Value of Metaphysical Belief

Questions about the value of metaphysical truth should also be distinguished from questions about the value of metaphysical *belief*. These might be questions about the value of having such a belief, independently of its truth or falsity:

(4) Will it be better (for us) if we believed that God exists?

Belief in God might be a source of psychological comfort whether or not God truly exists. It might have good consequences whether or not it is true or even justified.

That a belief has such an effect would often just be a fact about our psychology. In some cases, however, in order to explain the effect we would need to refer not just to the metaphysical belief but also to the evaluative significance that the believer attaches to its truth. The comfort of belief in God's existence is likely to be, not a brute psychological fact, but due to the great good that the believer associates with God's existence. It might reflect the believer's answer to questions such as (2).

Another type of question asks about the value of some metaphysical belief, *in light* of its truth or falsity:

(5) If God exists, will it be better (for us) if we believed that God exists?

Pascal's wager operates at this level: if God exists and will send us to heaven for believing in him, then that would be, for most of us, a desirable consequence of belief.

Here the tie between true belief and value is causal. But there are, as we already saw, more direct ways in which true belief (or knowledge) can be valuable: true belief about important matters—such as whether God exists—might simply be valuable in itself. Then there is the badness of error, especially of error that prevents us from appropriately responding to our reasons for action. Such error will often have many further negative consequences.^4^

However, although fundamental facts about the universe might be important, and thus valuable, whether or not they themselves make a difference in value, it seems plausible that facts that *do* make a significant difference in value are *more* valuable to know. If our answer to (2C) is that if God exists things would be dramatically better, then it will be more valuable to find the correct answer to (1). So again this question about the value of belief can interact with our question about the value of the truth of metaphysical views.

Questions such as (4) and (5) are questions about the value of metaphysical belief. Evaluative questions such as (2) are questions about the value of philosophical possibilities: questions not about whether it's good to believe that p is true, even whether belief that p would have good consequences, or be good in itself, if it's true, but about the value of *p itself*. These are questions about the world, and how it might be, not about us. Indeed these are questions that can often be asked about worlds in which we do not exist, even about worlds where no rational beings exist.

Of course, to the extent that the world we are considering does include us (or other rational beings), then the value of our beliefs might contribute to the total value of that world. So answers to questions about the value of belief, as well as (through the bad consequences of error) answers about normative implications, might also bear on our answers to questions about the value of metaphysical truth. Thus, although questions (1) through (5) are distinct, they can interact in multiple ways. Nevertheless, they should be kept apart. And very often, when we ask evaluative questions like (2C), what we are interested in, in the first instance, is in the direct evaluative difference made by some metaphysical difference, not on its impact on belief or error. We want to know whether the world will really be better if God exists, not about the miserable prospects of some proud atheist, or about the absurdity of religious faith in a Godless universe.

### The Psychology of Philosophy

It is not a secret that metaphysical belief is not always motivated solely by the disinterested pursuit of the truth. There are, for example, the familiar pressures to conform, the desire to be original, or even the sheer difficulty of admitting error. But deeper motivations can also influence metaphysical belief: a sense of what is at stake in a metaphysical dispute, a desire for the world to *be* a certain way.^5^ Some might, in this way, be led to believe in God or in the existence of objective values. But even a hard reductionist outlook might be rooted in ulterior desire.^6^

Philosophers might have such motives for metaphysical belief, both avowed and unconscious.^7^ But it's important to distinguish substantive questions about the value of philosophical possibilities from these questions in the psychology of philosophy. It *could* be that such underlying motives are shaped by evaluative intuitions or even explicit value beliefs that also happen to track the evaluative truth. In this way, the evaluative questions that concern us might partly overlap with these psychological questions. But the overlap is likely to be limited. Intuitions about value might be a shared starting point, but they are not likely to resemble the destination of evaluative inquiry.

Where there is such overlap, then the substantive evaluative inquiry might shed light on these psychological matters, suggesting possible biases in belief, biases that can have epistemic significance. If belief in God's existence is strongly shaped by the desire that He exists, and not by the evidence, this can count against the justification of that belief. But there is also the danger that the results of evaluative inquiry would themselves bias subsequent metaphysical belief—people might be less inclined to believe in a view that turns out to have especially bleak implications. And for these and further reasons, our answers to the evaluative questions might themselves be biased. As I noted, philosophers tend to want the metaphysical view they defend to also describe an appealing universe.

### Axiology as a Guide to Metaphysics

What is true is one thing, what we want, or take to be bad, another. If our preferences or value beliefs do influence our beliefs, this is an epistemic vice, something to resist. Wishful thinking is a constant danger, disinterested belief often an achievement.

There might, however, be ways in which value can imply or provide evidence for truth, ways in which axiology might be a guide to metaphysics. I do not have in mind here the link between value and truth that we find in some pragmatist theories of truth. Pragmatism ties the truth of a proposition to the value of *belief* in that proposition, not, incoherently, to the value of its truth.^8^ Nor do I have in mind a familiar form of argument that takes as its premise the truth of some normative claim and attempts to derive a metaphysical conclusion. For example, some argue that, given that we know that we have certain moral obligations, then, given that moral obligations would not hold if God did not exist, we must conclude that He does.^9^ Notice, however, that although those who put forward such moral arguments for the existence of God are clearly not indifferent to the prospect that morality is a myth, this valuation actually plays no role in the argument. If the premises of this argument were true, it would still go through even if it was, in fact, *far better* if morality was a myth.

But suppose that God did exist. God is held to be not only omnipotent and omniscient, but also supremely good. On one view, this implies that the actual world is the best of all possible worlds. Even on weaker views, it implies that the actual world is better than most alternatives—that if one possible universe is better than another, God is more likely to create the better one.

What follows from this is that if we do accept that God exists, we should accept at least some connection between value and metaphysical truth. Take for example libertarian free will. Theists sometimes hold that libertarian free will is better than compatibilist free will and that therefore God would endow us with the former rather than the latter.^10^ In such an argument, we move from an evaluative premise about the betterness of some possible world (or metaphysical property) to the conclusion that it in fact obtains (or is more likely to obtain). Notice that such arguments would also work in the opposite direction: if X exists, we can conclude that X is (or likely to be) in some way good, or at least contributes to making the world good overall. Here the actual is a guide to value. This is a familiar move in theodicies.

If God exists, then there is such a connection between value and metaphysical truth, and conclusions about the value of metaphysical possibilities could count as evidence for their actuality. Such a connection could also hold even if God doesn't exist. It would also hold, for example, on axiarchical views on which the universe we inhabit exists because it is better than the alternatives.^11^

If we do not see the world as shaped by value, then we would see no connection between the value of possibilities and their actuality. If anything, if some suggested view seems too rosy then we might take its claim to truth less seriously because it is likely to express wishful thinking rather than responsiveness to how things really are. It might be, as it's sometimes put, *too good to be true*.

However, when we ask whether some possible world is better than another, it might be useful, as a heuristic, to also ask which one God would (or should) choose.^12^ When theists argue that God would prefer libertarian to compatibilist freedom, they take themselves to be trying to figure out how things are. But even atheists can agree with the value judgment implicit in this theist claim. They can agree that it would have been better if we had such freedom, whether or not we actually do.

Whether this heuristic can really help us answer substantive evaluative questions is another matter (compare: finding out what we ought to do by asking what a virtuous person would do). And it has several limitations. It might be useful if we are asking about the impersonal value of possible worlds, not at all so if we are asking our question from a more partial perspective. And, of course, there is one key evaluative question we cannot use this heuristic to answer—the question whether it would be better that God exists. We can't intelligibly ask whether God would have created God.

## II. Philosophical Positions as Possibilities

Philosophers disagree about free will, consciousness, the metaphysics of morals, and the existence of God. In each such dispute, there are several contending philosophical positions (e.g. compatibilism, libertarianism, eliminativism, etc.), each of which claims to describe a metaphysical *possibility*—a way the world would turn out to be, if this position is correct. We can think of competing philosophical positions as describing possible worlds: worlds where God exists or does not, where we have libertarian free will or merely compatibilist free will or no free will at all, and so forth.^13^

Once this space of possible worlds is set out, we can ask what value these worlds possess, both absolutely and comparatively. If God exists, this might be in itself good, independently of any alternative. But God's existence might also be better than His inexistence. It might be better even if both possibilities would be good—indeed even if both are bad.

### The Problem of Impossibility

Philosophical positions describe opposing possibilities. We are interested in the value of these possibilities. In some cases, the possibility described is claimed to be contingent. Some identity theorists, for example, claim that the identity between conscious experience and brain states is merely contingent.^14^ These philosophers do not deny that substance dualism is a genuine possibility, and might have been true. But other philosophical positions make a stronger claim. They take themselves to be describing the way the world is *necessarily*. Many theists, for example, claim that God is a necessary being. On this view, atheism isn't just false, but couldn't be true. Some atheists return the favour. They claim that the concept of God is incoherent. They don't just deny that God exists, they deny He even could have existed.

When two opposing philosophical positions describe necessities and impossibilities in this way, then at least one of them does not describe a genuine possibility. They describe what are at most epistemic possibilities—ways in which things might turn out to be (even to necessarily be), *for all we know*. These epistemic possibilities will still be open to agnostics, or even to uncertain believers. But they will be closed to those who know that some position describes an impossibility—the possibility that God exists is closed to atheists who are certain that the concept of God is incoherent.

Some theists secretly worry that God might not really exist. Many agnostics fervently hope that He does. Thomas Nagel is one atheist who hopes that God *doesn't* exist.^15^ These are not random attitudes. They almost certainly express value claims, value claims about philosophical possibilities. But we just saw that on common forms of both theism and atheism, these might be value claims about *impossibilities* (or even about *impossible worlds*). But how could impossible worlds have value? To the extent that many philosophical positions turn out to describe such impossibilities, it might be that our evaluative questions have no answer—that theism, for example, does not really describe a genuine alternative, an alternative that might be good or better.

This presents a serious problem, though not, I believe (and hope), a fatal one. We should remind ourselves first that many philosophical positions make what are clearly contingent claims. With respect to other positions, it is not clear whether they are meant to state necessary truths. When Mackie claims that morality is an illusion, it is not clear whether he wants to presents this as a necessary truth, or merely the way things happen to be in the naturalist universe we inhabit. And other views, such as theism, are claimed to state necessary truths by some but not by others.

Now so long as we do not *know* that a philosophical position describes an impossibility, then it remains epistemically possible that this position is true, or at least that it could have been true. Here it seems that nothing prevents us from ascribing value to the possibility that this position appears to describe, at least on a provisional basis. After all, we see no problem about asking comparative evaluative questions about God's existence and inexistence when we consider Pascal's wager, or when we debate the problem of evil. We do so even though we are aware that, on some views, it might be impossible for God to exist—or not to exist. Since there are, I believe, only few philosophical positions whose mere possibility we can rule out with complete confidence, this leaves broad scope for evaluative inquiry into the value of the possibilities they describe.

Setting aside our epistemic limits, consider next the point that necessity, at least, is entirely compatible with value. Theists hold that God is perfectly good. Many of them hold that God is a necessary existent. But nobody holds that if God is a necessary existent, He *cannot* be good, or His existence could not be good. If anything, theists hold that the necessity of His existence lends *further* value to His goodness. God's existence is in this way claimed to contribute to the value of all possible worlds. Similar claims have been made about the value of Platonic forms and other abstract objects, or about the necessary order of the universe. And even complete atheists often speak about the beauty of some mathematical proofs. Thus, even when a philosophical dispute concerns necessities and impossibilities, at least one side of the dispute describes entities, properties or facts that can genuinely possess value, and add value to the world. There must be answers to at least some non-comparative questions about the value of the truth of certain metaphysical views.

Still, if impossibilities cannot possess value, then some of the ascriptions of value we want to make are merely apparent. If we came to know, for example, that it's impossible for agents to have libertarian freedom, we might need to withdraw any absolute and comparative value claims we earlier made about the possibility of such freedom. If we knew that God necessarily exists, we could value His existence, and, in one sense, see it as making the world better. But we could no longer hold that it would be bad or worse if He didn't exist.^16^

If this view is correct, then views and attitudes expressed by many theists (and, conversely, by many atheists) might not ultimately make sense. But it is not clear that we need to accept this view. Although many reject talk of impossible worlds and take counterpossible conditionals (which would be implicated in the ascription of value to impossibilities) to be all vacuously true,^17^ others have defended the contrary view.^18^ It certainly seems that to claim that that Golbach's conjecture is beautiful is to make a substantive, non-trivial value claim, a claim we could still make even if we knew that Goldbach's conjecture is mistaken (and thus necessarily false). And the claim that if God had existed, then, because God is omniscient, this would have the unappealing consequence that no one would enjoy complete privacy, remains an informative claim even for atheists who are confident that God couldn't exist.^19^

Finally, even if impossibilities are resistant to valuation, our evaluative *attitudes* might still find a legitimate target. Suppose, for example, that the concept of an omnipotent, omniscient and perfectly good being was incoherent. The existence of an immensely knowledgeable, powerful and benevolent being might still be a genuine metaphysical possibility, and value claims about theism could migrate to this adjacent possibility, allowing us to make rational sense of the attitudes associated with these value claims.

### Metaphysical Asymmetries

Still, if value really cannot be ascribed to impossibilities, this would set limits to axiological inquiry. It would therefore be useful to clarify the scope of this potential problem. Which philosophical positions are likely to be not just false but necessarily false?

Consider the dispute between libertarianism and compatibilism. Many compatibilists don't just think that we lack the kind of freedom that libertarians describe, or that such freedom is incompatible with naturalism, but that it's not even coherent. It would, however, be harder for libertarians to similarly deny that agents could exist who only enjoyed the kind of agency described by compatibilists. Libertarians, of course, would deny that such agents would be truly free, given that, on their view, such agency is not sufficient for freedom. Still, libertarians must surely concede that agents could exist who had such agency *and* employed, not a libertarian concept of freedom but the concept of freedom described by compatibilists. And to conceive the possibility that compatibilism is true is in essence to conceive *this* possibility. Thus, even if libertarianism is true, there would still be a good sense in which the alternative compatibilist world remains a possibility to which absolute and comparative value can be assigned.^20^

In this way, positions that are metaphysically more demanding would often allow for the coherence of less demanding alternatives, but these would not return the favour. If we hold that impossibilities cannot possess value, then this implies an intriguing asymmetry: if we inhabit a libertarian world, we can perhaps truly assert that libertarianism is better than compatibilism—but if we inhabit a compatibilist world, there could be no libertarian alternative, so this proposition might make no sense!

Such asymmetries suggest a general point. The axiology of metaphysics might presuppose controversial claims about modality and value. This could have an ironic consequence, because such claims are least likely to be compatible with hard naturalist views. The implication might be that if hard naturalist views are correct, then alternatives to them do not even make sense. Thus, even if we had strong reason to hope that such naturalist views are not true, because things would be far worse if they were, it might also be the case that if we knew them to be *in fact* true, we could not rationally regret this fact.^21^

### Which Possibilities should We Compare?

One worry, then, is that there are too few genuine philosophical possibilities for us to evaluate or compare. Another worry is that are *too many*.

Take the question whether it would be worse, as some think, if the freedom we have is compatibilist rather than libertarian. To answer this question, we need to compare compatibilist with libertarian worlds. But which worlds are we to compare exactly? It seems we can conceive an infinity of determinist worlds that contain agents with compatibilist freedom, worlds with different starting points and perhaps governed by different laws of nature. Things are not better when we turn to libertarian worlds. After all, with each choice situation, however banal, such worlds bifurcate into opposing possibilities, which continuously multiply. Or take atheist worlds. When we ask whether it would be bad if God doesn't exist, which world are we considering? Surely there is an infinity of possible ways in which things might unfold in God's absence.

It seems that philosophical positions delineate not specific possibilities but vast sets of possibilities, and it might make little sense to try to answer evaluative questions about philosophical possibility by comparing some arbitrary pair of worlds.^22^ Nor does it seem helpful (or practicable) to compare (if we could) the average value of each set, or to compare the best or worst world on each side. Our evaluative questions would be uninteresting if we can't find plausible constraints on the range of possibilities we are comparing.

It might be replied that, at least if we are comparing theism and atheism, there is a simple solution to this problem. Some theists would deny that there is an infinity of possible atheist worlds. They would claim that, if we can even conceive of such a world, what we must conceive is an empty void, containing no value. Then there are theists who would claim that there is also exactly one relevant theist world: the actual world, which also happens to be the best possible world—though most atheists would reply that, if anything, a theist universe should be far *better* than the actual universe, which, to put it mildly, contains much that could be improved.

These contentious claims are not useful solutions to our problem. But there is a better solution. When we ask whether it would be worse if God doesn't exist, this question can be naturally read as a question about the *actual* world. It compares one way in which things could turn out to be (God does exist) with another (He doesn't). These are two highly determinate worlds, sharing as much physics, history and biography as it is plausible to hold fixed. A similar approach seems appropriate even if we assume that one of the competing positions is in fact true. A theist can ask, for example, how bad things would be if his atheist opponents were in fact correct about the actual world, even though he is confident they are mistaken. Such a theist would be imagining a counterfactual atheist world that is as similar as possible to the actual theist world. Although here the evaluative question is asked from a different epistemic standpoint, it is essentially the very same question asked by the agnostic—it is a question about the comparative value of the same two possibilities.

I do not want to deny that sometimes it would be useful to compare the actual world, in which one philosophical position is assumed to hold, with a radically different counterfactual world. For example, atheists who consider whether God's existence would be better can legitimately conceive this counterfactual possibility as in many ways superior to the miserable actual world. But, for the reasons noted above, value claims that rely on such comparisons are likely to be contentious.

But let us return to the agnostic's question. I said the agnostic is comparing two highly determinate worlds—competing ways in which the actual world might turn out to be. But there are still a vast number of ways in which things might unfold on each alternative, ways that would make a dramatic difference to their value. And there is a whole range of issues we need to resolve, such as the question of the existence and nature of hell.

These are genuine problems. But they arise for virtually any interesting evaluative question of sufficient generality. (Compare: Which political system is best?^23^) On a common view of death's badness, for example, death is bad because it deprives us of possible good—a claim that similarly requires us to compare death with the numerous different ways in which a life might have gone on. If, by adding plausible further assumptions, we can still reach useful conclusions about these other questions, we should be able to do the same here.

In any case, the questions that interest us most will often be abstract enough to allow us to bracket this issue. We want to know what *direct* evaluative difference some metaphysical difference would make. For example, holding lives fixed, we might want to know whether a life would be better if a person had libertarian rather than compatibilist freedom. Indeed some hold that God would have reason to give his creatures libertarian freedom even if that freedom could then be exercised in ways that would make for an overall far worse world.^24^

## III. The Evaluative Framework

### Which Notion of Value?

Let us now set these difficulties aside. If we could ascribe value to philosophical possibilities, how are we to go about doing that?

In Pascal's wager, such questions are asked from the standpoint of a single person's good. Although we can ask such evaluative questions about philosophical possibilities, I have instead focused on their impersonal value.^25^ I asked whether it would be better *tout court* if God exists, or we have free will. This was a question that interested G. E. Moore:

By combining the results of Ethics as to what would be good or bad, with the conclusions of metaphysics, as to what kinds of things there are in the Universe, we get a means of answering the question whether the Universe is, on the whole, good or bad, and how good or bad, compared with what it might be…^26^

To answer such evaluative questions, we need to sum up the total value of some possibility and compare it with that of another. This procedure is familiar from consequentialism, but our question is not at all concerned with the consequences of possible acts. Nor is it a question about what is good from the standpoint *of* the universe, to use Sidgwick's phrase, since we are considering the value of *alternative* universes. It might be better to speak of God's standpoint, the standpoint of someone who could choose to create one of these alternatives. Even this would be slightly misleading, since God's existence is itself one possibility we might want to consider.

There are those who doubt that we can intelligibly assess the total value of worlds.^27^ But even these philosophers could still ask a closely related question: they can ask whether some metaphysical difference would make things better or worse in some *respect*. And, as noted above, this narrower question will often be our primary concern. It will often be difficult, even impossible, to assess the total evaluative impact made by some metaphysical difference, a task that might require us to track numerous causal consequences. But the question of the *direct* difference in value made by a given metaphysical difference seems tractable.

### Evaluative Constraints

To ascribe value to possible worlds we need some evaluative standard, and which standard could we employ, if not our own? Since there are great differences in evaluative outlook, it is not likely that we shall always arrive at the same answers. Disagreement about such answers, however, is simply substantive evaluative disagreement, disagreement about what matters. And this disagreement should be intelligible independently of the philosophical dispute we are considering.

Things, however, get more complicated when some of the philosophical possibilities under consideration *exclude* or *imply* certain evaluative claims. Suppose, for example, that we ask whether it would be better or worse if morality turned out to be merely an illusion.^28^ In answering this question, we need to conceive of a world where there is *nothing* wrong about sadistically torturing or killing innocents. It makes no sense to respond to this possibility with *moral* horror. For to conceive of the truth of moral error theory is precisely to conceive of a world that contains *no* moral value—such value is simply *excluded* from this possibility and cannot be used to asses it. Our beliefs about moral value need to be bracketed when we consider the value of an amoral world.

There is a more extreme example. Some people fear that nothing really matters. But such angst about evaluative nihilism also makes no sense. A nihilist world is not simply one containing *no* value or possessing *zero* value. Nihilism describes a world in which there is *no such thing* as value. So we cannot coherently say that the truth of nihilism would be a bad outcome.^29^

Does this mean that when we compare the value of two philosophical possibilities, we can only legitimately appeal to evaluative standards that can be applied to both—that, for example, when we compare the value of moral error theory and of moral realism, we must bracket moral value, and compare only the *non-moral* value of the two alternatives?

That would also be a mistake. To see this, consider theism. Since the traditional concept of God simply *entails* that God is supremely good, then when we are asking whether it would be good or better if God exists, we need to consider a world which on the face of it contains more good—God's own goodness. And, given that God is also omnipotent and omniscient, He can also be expected to produce much further good. Since an atheist world would lack this value, it seems at least prima facie worse. So you might think that the evaluative question is tilted in favour of theism. But this is not unfair, let alone incoherent. To ask whether God's existence would be better is *precisely* to consider a possibility which contains a being who is supremely good and powerful in these ways. This is simply definitional of the possibility under consideration. And recall that we are considering here a question about value, not about truth. Indeed, atheists might want to claim that a theist world seems much better precisely *because* it is a projection of our deepest wishes. (Some views win the betterness contest precisely by losing the truth contest.)^30^

Similar claims might apply in the case of morality. Now it is definitional of morality that many *normative* claims are true in a world where morality isn't an illusion. Not everyone would agree that morality also inherently involves a range of distinctive *value* claims, but to the extent that it does, it would be similarly legitimate to add this moral value to the total tally of a moral world. Thus although sacrificing oneself to help a stranger would not be good in itself in an amoral world, it *would* be a great good in a moral world, and make it a better world.^31^

Things are less clear, however, when we consider claims about value that are associated with certain possibilities only in a looser sense. Theists might associate theism not only with a definitional claim about God's goodness, but also with *substantive* value claims that would be rejected by many atheists—claims, for example, about the *spiritual* value of certain religious practices and rituals, or of religious experience. If we accept these value claims, the theist world might be even better.

When we consider the value of a theist world, it isn't clear that the world we are contemplating also needs to be one in which these substantive value claims are true, if they cannot be derived from value claims such as God's goodness, which are constitutive of the possibility in question. However, we *would* have reason to take these value claims into account if they are ultimately grounded in other value claims that we do accept. For example, the value of certain religious rituals or experiences might be explained as deriving from the value of being in an appropriate relation with God, which in turn could be explained as a unique instance of the theism-neutral value of relations between persons. In this way, an atheist could agree that, if God did exist, certain attitudes and acts that express our relationship to Him would be of great value.

Whether or not such a strategy could be made to work, it seems clear enough that at least *some* substantive value claims that are often associated with theism *could* be bracketed. Most theists are also believers in some religion, belief that typically involves commitment to a range of substantive value claims. But if, as agnostics, we ask whether it would be better if theism turned out to be true, we need not be asking whether, for example, *Christianity* turns out to be true—where this includes assuming the truth of a range of value claims we do not, in fact, now accept. That the truth of Christianity would be better than its falsity when this question is considered from a Christian evaluative outlook is not terribly surprising, or interesting.^32^

## IV. Evaluating Philosophical Possibilities

### Disagreement about the Existence of Facts, Entities or Properties

One familiar kind of philosophical disagreement is over metaphysical questions such as

(1) Does God exist?

Many theists think it would be both far worse, and absolutely bad, if God did not exist. Many similarly think that it would be awful if free will turned out to be an illusion, or if morality is merely a myth.^33^ These are value claims about a particular kind of philosophical possibility—variously called error theory, nihilism or eliminativism regarding certain facts, entities or properties.

Would it really be worse if God does not exist, or if morality is merely a myth? To answer such questions, we need to determine the value that these facts, entities and properties contribute to a world. In the case of theism, this is relatively straightforward. God is defined as possessing supreme goodness, and as the source of much further good. In most other cases, the connection to value is not definitional, but dependent on substantive value claims. These would have evaluative implications, when conjoined with metaphysical claims, in the same way that they have such implications when conjoined with empirical claims. For example, if we hold that

(6) Freedom of the will is intrinsically valuable

and it is an empirical truth that

(7) No one has free will

whether because all life in the universe has been extinguished, or because we are considering a universe that is inhospitable for life, then the world we are conceiving would contain, in this respect, less value than the actual world which contains billions of free persons.

The same should also follow, however, if (7) was a metaphysical claim that is true, say, because our concept of freedom is libertarian but determinism is true. Here we would be comparing, not an empty universe with ours, but two ways in which our universe could turn out to be: one in which it contains billions of free persons, as we now believe, and another where this belief is mistaken.

There might, however, be an important difference between these two types of cases. To the extent that the metaphysical variant of (7) amounts to the claim that no one *could* be free, or even that the concept of freedom is incoherent, then it might be doubted if (6) could even be said to be true. After all, we have come to believe in this value claim through reflection on our concept of freedom, reflection that now seems to have been based on a metaphysical mistake, or to even lack a genuine target.

However, as we saw earlier, this needn't present a problem. We can legitimately appeal to value claims that might be strictly compatible only with one of the two philosophical possibilities. In any case, even if in the eliminativist world where (7) is true because freedom is incoherent, freedom couldn't even be said to be valuable, the end outcome would still be the same as if freedom *was* valuable but never instantiated. So this difference makes no evaluative difference.

### Disagreement about the Nature of Facts, Entities or Properties

Atheists believe that God does not exist, and that we should just move on. But Mackie and other moral error-theorists believe that although our moral discourse is in error, moral practice should continue as before—now understood in antirealist terms. This is a claim about the (non-moral) value of moral *belief* (or of moral practice), not about the value of a philosophical possibility.^34^ But when such claims are plausible, then comparing a world in which moral realism turns out to be true and a world where the error theory is true but we nevertheless go on as before might not be very different from comparing the truth of moral realism and that of moral antirealism.

This takes us to a second, and more common kind of philosophical disagreement, that between competing accounts of some discourse or concept. An example would be

(8) Assuming we do have free will, which view gives the correct account of our concept of free will, libertarianism or compatibilism?

In this type of philosophical disagreement, all the disputants agree that at least some of the positive propositions of the discourse are true (or that the relevant facts, entities or properties exist), but disagree about what it is we are saying when we assert these propositions (or about the nature of these facts, entities or properties).

Could the outcome of this kind of dispute make an interesting difference in value? Here the competing philosophical views all attempt to preserve the truth of the first-order propositions of the discourse, and this would presumably mean that they would also preserve the truth of the substantive evaluative propositions that apply to them. If we possess freedom, and freedom is valuable, then we should have this value on whichever account of freedom turns out to be the true one.

It certainly seems to be a common view that there is far less at stake in philosophical debates between competing accounts of a discourse that do not involve the imputation of error. Some ethicists think, for example, that, since the outcome of disputes between competing realist and antirealist metaethical views of a sophisticated enough sort would make no difference to the truth of substantive normative claims, such metaethical disputes are of little interest.^35^ If something like this view is correct, parallel disputes in other areas of philosophy would also turn out to be relatively unimportant.

But this conclusion is premature. For one thing, although competing accounts of some region of discourse typically aim to preserve the truth values of *paradigmatic* first-order propositions, they often have opposing implications for the truth values of at least *some* propositions. This is clearest when such disputes revolve around thought experiments alleged to reveal the counterintuitive implications of some view. And these differences in first-order truth might in turn make an evaluative difference. However, to the extent that the first-order divergence between such competing accounts only occurs in extremely far-fetched cases, then the evaluative difference they generate is likely to be negligible. Worse, this first-order divergence is often itself subject to heated dispute. Proponents of philosophical views often deny that they have the alleged counterintuitive implications. It is thus unclear then whether in conceiving how things would be if some philosophical view was true we should also include such disputed implications.

There are, however, areas where the first-order divergence in the actual world appears substantial. For example, although competing accounts of consciousness typically aim to preserve everyday ascriptions of consciousness to adult humans, some imply that many animals cannot be conscious,^36^ whereas others either rule out or easily allow for conscious machines. Then, of course, there is the example of the dispute over whether embryos are persons.

Recall that we are not asking about the normative implications of such disputes. The normative implications of the claim that embryos are persons have been much discussed. We are asking whether it would be *better* or *worse* if embryos were persons. To the extent that the moral implication of this claim is that abortion is a serious moral wrong, then a world where embryos are persons and abortion is widely practiced would be plainly a *far worse* world than one where embryos are not persons. However, once we bracket the question of error and its implications, it is far harder to say whether it would be better if we become persons at conception or only at some later point. As noted above, metaphysical differences that have momentous normative implications may nevertheless make little or no evaluative difference.

### Metaphysical Values

When philosophical positions diverge in these ways in their first-order implications, the evaluative difference that this makes depends on substantive evaluative claims of a familiar kind. Such divergence can affect the scope of the application of these claims.

But suppose that two opposing philosophical accounts of a discourse agreed (or were neutral) with respect to all the first-order claims of that discourse. Could their truth or falsity still make a significant evaluative difference? I believe it can.

This comes out most clearly when we consider noncognitivist or irrealist views. Such views are a special case. Like error theories, they in one sense deny that the relevant facts, entities or properties exist. But they also claim to fully preserve the first-order discourse, understood not as stating plain truths but, for example, as expressing some non-cognitive mental state. To start with an extreme example, consider the evaluative question,

(9) Will it be better if God exists in the sense defended by robustly realist theists, or in the sense defended by irrealist theists?

Although we can correctly assert that God exists on both views, they clearly describe utterly different worlds. In one there is a metaphysically separate supernatural entity, a benevolent creator with unlimited causal powers. In the other there is no such entity, only a distinctive range of experiences and attitudes that are expressed in the context of certain religious practices. Of course, irrealist theists would deny that on their view God does not *really* exist, and they typically hold that their realist opponents misunderstand religious discourse. But now we are asking them to consider how things would be if they are *mistaken* about this. I strongly suspect that most would agree that the world described by realist theism is better than that described by irrealist theism. This example is somewhat complicated by the fact that in a world where a realist God exists there might literally be an afterlife and virtue would really be rewarded, which might be more appealing than the merely expressive or metaphorical counterparts of these goods.^37^ But I suspect that a realist theist world would strike most of us as superior to an irrealist alternative even if we bracket this point.^38^

It seems to me generally true that when we compare robustly realist views of some domain of discourse with competing accounts, both noncognitivist and cognitivist, then—to the extent that the facts or properties in question are taken to possess positive value—the worlds described by robustly realist views seem, in one important respect, better.^39^ The world described by moral realism, for example, seems to me clearly better than the one described by moral noncognitivism. It is not easy to articulate the values that underlie this preference for facts, entities and properties that are robustly real or even *sui generis*. Since these value claims do not relate to differences in the truth of first-order propositions, they must apply to differences in their *content*. That is, they must apply to what we might call philosophical properties: properties such as mind-independence and irreducibility. These values apply not to the property of possessing freedom of the will, but to properties *of* that property, as set out by some philosophical view. Such value claims thus concern what we can call *metaphysical* values.

Consider an example. On some ways of understanding libertarian freedom or qualia, these would be either sui generis non-natural properties, or just brute fundamental natural properties. On either view, freedom and consciousness are singular irreducible attributes of human beings, whereas on competing compatibilist or functionalist accounts, they are constituted by complex neural patterns which are entirely continuous with the rest of the physical world. If uniqueness and singularity endow things with value, free will and consciousness would be more valuable on these non-reductionist views, making us special entities in which fundamental aspects of reality uniquely inhere.

These are just meant as examples of metaphysical value, and others might be attracted to a contrary view on which it can better that some entities or properties *are* dependent on, or even created by us. (Though think of common reactions to the Experience Machine—and of the very limited attractions of solipsism.)^40^

Moreover, there might also be metaphysical values that apply to global features of a world, and these might pull in a different direction. Thus, although it might be better if we were Cartesian egos, and thus unique, simple and irreducible entities, this might also be worse in a global sense, given that a monist universe in which everything is reducible to a single substance might be a more unified universe. A strongly reductionist naturalist view might thus still describe a world that is overall more valuable.

Notice that although unity and simplicity are often seen as theoretical virtues, here we are not asking whether simpler *theories* are better because more likely to be *true*, but whether simpler *worlds* are better *in themselves*—whether, for example, God would have reason to prefer a simpler and more unified world to messier alternatives. This is a substantive value claim that some would deny.^41^ My aim here was not to defend any of these value claims, only to draw attention to the overlooked point that philosophical properties can be valued and disvalued just like any other aspect of the world.

### What Evaluative Difference can Metaphysical Values make?

I have argued that the worlds described by competing philosophical accounts of a discourse can still differ in value even if they agree on the truth values of its first-order propositions. This suggestion needs to be unpacked.

Suppose that libertarian freedom is, in this way, more valuable than compatibilist freedom. This could simply mean that when we compare libertarian and compatibilist worlds, we would have stronger reason to prefer the libertarian one. The metaphysical difference generates a difference in the value *of* the world, but makes no difference to value *within* the world. But a stronger view also seems plausible. On this stronger view, the freedom we would possess in the libertarian world would be worth *more* than that we would possess in the compatibilist one. This would be a difference in value *within* worlds. Although in both worlds, the possession of freedom might ground a distinctive moral value or status, the difference in metaphysical value could make a difference to how this value is *weighed* against other values—in a libertarian world, the value of freedom might have greater weight. Differences in metaphysical value could thus lead to significant normative implications. This does not strikes me as implausible—indeed it seems to me that when philosophers accept a reductive view of some phenomenon, they are often also led to think of that phenomenon as possessing less value.

There is one case, however, where it is hard to see how such differences in value could be manifest within a world. I suspect that many would agree that

(10) It will be better if value was an objective, mind-independent property, than if value was grounded in our subjective responses

If this is true, we would have reason to prefer a value objectivist world to a value subjectivist one. The truth of value claims such as (10) is entirely compatible with the truth of subjectivism—it might be that what we want after vivid reflection on the relevant facts is precisely that things would possess value *independently* of our desires. What is doubtful is that the truth of such claims could make a difference *within* worlds. How could goodness be *more good* in an objectivist world? Although Nietzsche wrote about the re-valuation of all value, the idea that value could be generally more valuable makes no sense.

## V. Our Attitude to the Universe

Questions about the value of philosophical possibilities are not questions about what we would have reason to do, if some philosophical possibility obtained. They are not, in this sense, practical questions. This might make them seem pointless. Even if some philosophical possibility was far better than another, why should we care? Such differences in value seem to matter little. They seem to matter even less if they refer to very distant possibilities, let alone to known impossibilities.

We are typically interested in the value of possibilities as a guide to action. We want to bring about the better outcome. With respect to philosophical possibilities, this is almost never an intelligible aim. We cannot aim to create God, if He does not exist. Nor can we hope to generate a mental substance, or libertarian freedom, in the laboratory. If God exists, or does not, that's just the way things are, perhaps necessarily.

Action is important, but it isn't everything. It often matters greatly how things are, or how they might have been, even if there is nothing we can do about it. We care about the past, and not only as a source of lessons for the future. And if value mattered only as a guide to action, then, absurdly, believers would have no reason to value the goodness of God's existence, only to draw its practical implications.

If some possibility is good, or better, this gives us reason to have certain attitudes to it. If, as some think, libertarian freedom is better than compatibilist freedom, then we have reasons to *prefer* a libertarian world to a compatibilist one—to hope that libertarianism would turn out to be true. If it would be extremely bad if it turned out that we are not free, then this is a possibility we have reason to fear. And if we believe that we are in fact not free, this might be something to deeply regret.

If we know that God exists, and that He exists necessarily, then perhaps we cannot rationally prefer that He exists. It might make even less sense to prefer that God not exist, if He must. But there are other attitudes we can have towards the necessary—perhaps even towards the impossible. We can, for example, take pleasure in God's existence, or be displeased by it.

It might be objected that even if there is a sense in which, if some philosophical possibility would be better, we have reason to prefer it, this still does not show that this matters much. After all there is an infinity of ways in which things might be better—or worse. There are numerous utopias and dystopias we could conceive. But it would be a pointless exercise in axiological accountancy to add up their value, or to make them the objects of our hopes and fears. Such daydreaming might even be a vice.

In many contexts, whether some possibility commands our attention depends not only on the difference in value that it would make, but on its probability. We have little reason to be concerned about possibilities that have negligible chances of being realized, even if they would make things far better. Thus, although questions about God's existence, or about libertarian freedom, might be vital questions to the agnostic, they might be of far less concern to those who are confident that God does not exist, or that libertarian freedom is a fanciful illusion. But sometimes we have reasons to care deeply even about possibilities that are not within reach—about possibilities which were never in the offing. For example, even if it is perfectly clear that we inhabit a Godless naturalist world, we would still have strong reasons to lament God's nonexistence if we also believed that, because of His absence, human life lacks meaning.

In these ways, we can have reasons to care deeply about different philosophical possibilities and their value. These are not just reasons to feel this or that. They are also reasons to try to answer certain philosophical questions—reasons whose urgency should reflect how much is at stake in these questions. And although we cannot have reasons to try to realize the impracticable (let alone the impossible), we can have reasons to act in ways that express our deepest attitudes to the way things are, or might have been. Evaluative questions about philosophical possibility may, in this way, have a profound impact on the way we live our lives. If this claim seems surprising, reflect for a moment on the history of art, literature and culture in the last three hundred years. It is impossible to fully understand this history except as a response to dramatic upheavals in metaphysical belief.

Some past philosophers have held that our greatest calling is to engage in joyous contemplation of God or the order of the universe. This may not be our greatest calling, and there may be no divine order to admire. There is nevertheless an imperative to comprehend the fundamental nature of the universe, and to respond to it with appropriate attention and feeling, a task that requires us to contrast our world with conceivable alternatives, both better and worse. This is not daydreaming. On the contrary, it would be a kind of practical solipsism to be indifferent to the vast totality we inhabit, and to be concerned only about the causal effects we can produce using our laughably limited agency.^42^

## Footnotes

1 Questions about value are sometime discussed in the free will debate, though typically only in connection with a defence of specific metaphysical views. See for example, Dennett, 1984 and Smilansky, 2010. For an explicit discussion of evaluative questions about free will, see Haji, 2009. I consider such questions about God's existence in Kahane, 2011; see also McTaggart, 1909; Rescher, 1990.

2 Answers to such evaluative questions might also have implications for the epistemology of philosophy: if knowledge requires meeting more stringent epistemic standards when the practical stakes are higher (Stanley, 2005), then arguably this should also hold when the *evaluative* stakes are higher.

3 On some views, evaluative claims can be reduced to normative ones. But the distinction still holds: it would now be a distinction between what we have reason to *do* within some world, and our reasons for *attitudes* towards that world. I discuss such attitudes below.

4 We mustn't, however, confuse the badness of error with that of genuine loss. We cannot lose what we never really had. Talk about discovering that ‘God is dead’ is, in this way, highly misleading.

5 William James notoriously suggested that “[t]he history of philosophy is to a great extent that of a certain clash of human temperaments” (James, 1907/2000, 8). Fichte and Nietzsche make similar remarks.

6 Cf. Nagel, 2001, p. 130; van Inwagen, 2009, pp. 203–205. Nietzsche even speculates that belief in exceptionless natural laws is driven by ‘plebeian antagonism’ to privilege (Nietzsche, 1886/1966, p. 30).

7 For an interesting application, see Nichols, 2007.

8 The same goes for views such as Carnap's, which see competing metaphysical views as describing alternative conceptual schemes which cannot be said to be true or false, and between which we must decide on pragmatic grounds. (Contrast Pascal's wager, where (prudential) value is a pragmatic guide to belief in a proposition with an *independent* truth-value.)

9 Adams, 1979; Zagzebski, 1987.

10 van Inwagen, 2006.

11 Leslie, 1979; Rescher, 2000.

12 This would be more than a heuristic on views on which to be a better universe simply is to be the universe that would be preferred by an ideal observer.

13 I will use ‘possibility’ and ‘possible world’ interchangeably, but nothing I say requires this way of understanding modality.

14 Armstrong, 1968.

15 Nagel, 2001, p. 130.

16 Could we have reason to prefer one of two epistemic possibilities, if we also believe that only one of them is a genuine metaphysical possibility? Suppose we believe that if p, things would be pretty good, and that if not-p, they would be rather miserable—and that if one of these holds, it holds necessarily. Can we rationally prefer p to not-p? Can we prefer it while also thinking that if it holds, it's not *better* than any alternative—because *there is none*? There are obvious echoes here of the non-identity problem.

17 Stalnaker, 2003, ch. 3; Williamson, 2007, ch. 5.

18 Nolan, 1997; Brogaard & Salerno, 2007; Yagisawa, 2010, ch. 8; Zagzebski, 1990. Nolan presses the general philosophical price of rejecting counterpossible reasoning. My discussion suggests a further price: that many common and seemingly intelligible attitudes and evaluations may no longer make sense. Though notice that even if we could intelligibly ascribe value to impossibilities, this wouldn't yet show that we should *care* about such impossibilities.

19 Perhaps we cannot make sense of ascriptions of value to what are clearly *logical* impossibilities—perhaps there is really nothing here to which value could attach; but not many serious philosophical views are simply incoherent. And few would deny that we can ascribe value to and compare *physical* impossibilities, let alone merely *practical* ones. What is not clear is whether we can genuinely ascribe absolute and comparative value to *metaphysical* impossibilities. But why should metaphysical impossibilities be more problematic, in this respect, than physical impossibilities? (I benefited here from discussion with Tim Mawson.)

20 It might be objected that, if concepts are individuated by their content, then if our concept of freedom *is* libertarian then this alternative simply isn't intelligible. Notice first that even libertarians can accept that there are senses of the word ‘freedom’ that are compatibilist in character (Kane, 1994, pp. 29–30). But even if our discourse about freedom was exclusively libertarian, we could still coherently ask whether libertarian freedom is better than the kind of agency described by compatibilists—we could still wonder whether it would be worse to possess agency only in this sense, even if it wasn't the referent of our actual concept of freedom. Our evaluative questions are about properties and facts, not concepts.

21 There are, however, important exceptions to this asymmetry. If God exists in a robust realist sense, and exists necessarily, He would of course still exist even in a world where no one believes in God, or where people have only an irrealist concept of God. Similarly, if objective values exist, then, on many views, fundamental value claims would state necessary truths, meaning that various things would be objectively good and bad even in worlds where evaluative concepts are subjective (see Kahane, forthcoming). In these cases, the truth of views that are *more* metaphysically demanding *would* rule out the coherence of weaker alternatives: if God exists necessarily, then the Godless world described by atheism wouldn't be a genuine possibility.

22 This problem echoes the many-gods objection to Pascal's wager.

23 Nozick, 1974, pp. 4–6. I am grateful to an anonymous referee for this reference.

24 van Inwagen, 2006, pp. 72, 90. Kierkegaard held a similar view. Cf. Jackson, 1998.

25 Haji, 2009 considers both kinds of questions.

26 Moore, 1953, p. 40.

27 Foot, 1985. Though notice that substantive questions about the impersonal (or personal) value of possible worlds don't commit us to any specific *metaethical* position—they can be intelligibly asked by realists, subjectivists and noncognitivists.

28 Nietzsche argued that morality is a pernicious illusion—he held both that morality is an illusion *and* that moral practice has pernicious consequences. He did not consider the possibility raised by my question: the possibility that morality *is* genuine—*and* that this is a pernicious outcome.

29 Although evaluative nihilism couldn't be bad, might it be *worse* than alternative worlds? Now *if* nihilism is true, we cannot coherently ask this (or any other) evaluative question. But what if it's false? Couldn't we assert, from within a moderately good world, that a world in which nothing is good or bad is worse (even though, if we *had* inhabited such a world, this *couldn't* be worse)? I suspect that such claims don't ultimately make sense.

30 This is almost the flipside of the problem of evil: the same considerations that make some view more likely to be *false*, given how things *actually* are, can also make it more likely to be *better*, *if* true.

31 There is however a further complication. It could be argued that when we ask whether it would be worse if morality was an illusion, this question must be asked from a personal standpoint, because to ask about the *impersonal* value of an amoral world is already to adopt something close to a moral standpoint. If so, then moral value would make a difference only if it affects prudential value.

32 When atheists ask whether it would be good if God exists, they needn't accept any of the substantive value claims associated with some theist religion. But can theists assume their truth when asking this question? This seems problematic when the source of these claims is revelation. However, this may not matter if we think of revelation as an instance of especially reliable testimony about truths that would hold even if the attester hadn't existed. Things are more hazardous when the attester is also the *ground* of these truths.

33 The young Fichte wrote: “I have been living in a new world ever since reading the *Critique of Practical Reason*…Things have been proven to me which I thought could never be proven— for example, the concept of absolute freedom, the concept of duty, etc.—and I feel all the happier for it. It is unbelievable how much respect for mankind and how much strength this system gives us.” (Fichte, 2005, 153)

34 These two questions might overlap: the non-moral value of the reality of morality will in part depend on the value of adherence to it. But morality itself can also be subject to valuation: just as morality has a dim view of some aspects of self-interest, self-interest might, as Nietzsche illustrates, have a dim view of morality.

35 Hurka, 2006.

36 Carruthers, 1992.

37 Noncognitivism presents another problem. In a realist theist world, God exists in some strong sense. In an noncognitivist theist world, God doesn't really exist, though we can correctly assert that God exists. How do we describe such a world? How do we represent the expressivist sense in which God *can* be said to exist? And if God is said to exist in such a world, and to be supremely good, do we add that value to the value or the world in the same way we add the value of God's goodness in the realist world?

38 I discuss this example in Kahane, forthcoming.

39 Greater reality thus *amplifies* value: it would make things *worse* if the fact/property in question is one we *disvalue*. For example, it seems to me that pain wouldn't be as bad if it were really just a pattern of behavioural responses, as some antirealists have thought. I am grateful to an anonymous reviewer for suggesting this example.

40 After Bertrand Russell abandoned his earlier Platonism and came to embrace an antirealist view of mathematics, he wrote that “[m]athematics has ceased to seem to me non-human in its subject matter. I have come to believe, though very reluctantly, that it consists of tautologies... I can no longer find any mystical satisfaction in the contemplation of mathematical truth.” (Russell, 1959, p. 212). Russell had a similar distaste for idealism and pragmatism (which he rejected). See Russell, 1919/1959, p. 159; Russell, 1969, p. 222–223.

41 It is denied by Cartwright, forthcoming. Cartwright reasons from her belief that the universe is not governed by exceptionless natural laws, conjoined with a theist assumption, to the conclusion that a messier universe must be better—a value claim she also seems to find attractive on independent grounds.

42 I am very grateful to Nicholas Shackel, Timothy Chan, S. Matthew Liao, Nick Bostrom, Tim Mawson and an anonymous reviewer for extremely useful comments. An earlier version of this paper was circulated under the title ‘Value and Philosophical Possibility’. Work on this paper was partly supported by a University Award from the Wellcome Trust (WTO87208MF).

## References

[b1] Adams RM, Delaney C (1979). “Moral Arguments for Theistic Belief”. Rationality and Religious Belief.

[b2] Armstrong DM (1968). A Materialist Theory of the Mind.

[b3] Brogaard B, Salerno J (2007). “Why counterpossibles are non-trivial”. The Reasoner.

[b4] Carruthers P (1992). The Animals Issue.

[b5] Cartwright ND, Bersanelli M, van Inwagen P, Harper C “God's order, man's order, and the order of nature”. Science, Reason and Truth.

[b6] Fichte JG, Breazeale Daniel (2005). Early Philosophical Writings.

[b7] Foot P (1985). “Utilitarianism and the virtues”. Mind.

[b8] Jackson TP, Hannay A, Marino GD (1998). “Arminian edification: Kierkegaard on grace and free will”. The Cambridge Companion to Kierkegaard.

[b9] Haji I (2009). “Incompatibilism's threat to worldly value”. Philosophy and Phenomenological Research.

[b10] Hurka T, Leiter B (2006). “Normative ethics: back to the future”. The Future for Philosophy.

[b11] James W (1907). Pragmatism and Other Writing.

[b12] Kahane G (2011). “Should We Want God To Exist?”. Philosophy and Phenomenological Research.

[b13] Kahane G “Must Metaethical Realism Make a Semantic Claim?”. The Journal of Moral Philosophy.

[b14] Kane R (1994). “Free Will: The Elusive Ideal”. Philosophical Studies.

[b15] Leslie J (1979). Value and Existence.

[b16] Moore GE (1953). Some Main Problems of Philosophy.

[b17] Nagel T (2001). The Last Word.

[b18] Nichols S (2007). “The Rise of Compatibilism: A Case Study in the Quantitative History of Philosophy”. Midwest Studies in Philosophy.

[b19] Nietzsche F (1886). Beyond Good and Evil.

[b20] Nolan D (1997). “Impossible worlds: A modest approach”. Notre Dame Journal for Formal Logic.

[b21] Nozick R (1974). Anarchy, State and Utopia.

[b22] Rescher N (2000). “Optimalism and axiological metaphysics”. The Review of Metaphysics.

[b23] Russell B (1912). The Problems of Philosophy.

[b24] Russell B (1959). My Personal Development.

[b25] Russell B (1969). The Autobiography of Bertrand Russell: The Middle Years.

[b26] Smilansky S, Campbell JK, O'Rourke M, Silverstein HS (2010). “Free Will: Some Bad News”. Action, Ethics, And Responsibility.

[b27] Stalnaker R (2003). Ways a World Might Be.

[b28] Stanley J (2005). Knowledge and Practical Interests.

[b29] van Inwagen P (2006). The Problem of Evil.

[b30] van Inwagen P (2009). Metaphysics.

[b31] Williamson T (2007). The Philosophy of Philosophy.

[b32] Yagisawa T (2010). Worlds and Individuals, Possible and Otherwise.

[b33] Zagzebski L (1987). “Does Ethics Need God?”. Faith and Philosophy.

[b34] Zagzebski L, Beaty M (1990). “What if the impossible had been actual?”. Christian Theism and the Problems of Philosophy.

